# Zn-MOF hydrogel: regulation of ROS-mediated inflammatory microenvironment for treatment of atopic dermatitis

**DOI:** 10.1186/s12951-023-01924-0

**Published:** 2023-05-22

**Authors:** Lirong Qiu, Chengcheng Ouyang, Wei Zhang, Jia Liu, Luting Yu, Guoguang Chen, Lili Ren

**Affiliations:** School of Pharmacy, Nanjing Tech University, 30Th South Puzhu Road, Nanjing, 211816 China

**Keywords:** Atopic dermatitis, Hydrogel, ZIF-8 nanoparticles, Inflammatory skin diseases, Antibacterial

## Abstract

Atopic dermatitis (AD) is a chronic and recurrent inflammation disease associated with immune dysfunction. The high level of reactive oxygen species (ROS) causes high oxidative stress and further results in the deterioration of AD. At the same time, the ROS produced by bacterial infection can further aggravate AD. Here, the prepared PVA-based hydrogel (Gel) has a high ROS scavenging ability, and the antibacterial agent Zn-MOF(ZIF-8) loaded into the hydrogel shows a lasting and effective antibacterial activity. Thus, a Zn-MOF hydrogel (Gel@ZIF-8) is prepared to regulate ROS-mediated inflammatory microenvironment. In vitro experiments show that Gel@ZIF-8 has good antibacterial effect and cell biocompatibility. In the AD-induced mouse model, Gel@ZIF-8 can significantly enhance the therapeutic effect, such as reduce the thickness of epidermis, the number of mast cells and IgE antibodies. The results indicate that the ROS-scavenging hydrogel could treat the AD by regulating the inflammatory microenvironment, providing a promising treatment for managing AD.

**Supplementary Information:**

The online version contains supplementary material available at 10.1186/s12951-023-01924-0.

## Introduction

Atopic dermatitis (AD), also known as atopic eczema, is a chronic and recurrent disease characterized by inflammation and itch [1–3]. Due to continuous scratching, allergens and microorganisms can easily penetrate into the deep layer of the skin, further causing imbalance of the epidermal microenvironment. After allergens in the environment enter the human body, they are captured by Langerhans cells (LC) and transferred to T cells after a series of processing [4, 5]. In the acute phase of the disease, most Th0 cells are induced to Th2 cells and express T helper 2(Th2) cytokines, such as interleukin (IL)-4, IL-5 and IL-13 [6]. B cells differentiate into plasma cells and secrete functional immunoglobulin E (IgE) through high-frequency somatic mutation and antibody type conversion [7]. After IgE receptor (FcεRI) on mast cell combines with IgE, it stimulates mast cell granules to fall off and release histamine, cytokines and chemokines, further accelerating the development of atopic dermatitis [8]. Steroids, antihistamines and antibiotics are usually used for treatment, but they will cause adverse side effects for a long time, such as hyperglycemia, poor wound healing, Cushing's syndrome and sleep disorders [9–12]. Therefore, it is necessary to find alternative therapies for AD.

Recently, the development of AD is considered to be related to excessive oxidative stress [13, 14]. Reactive oxygen species (ROS) at the normal level act as an important second messenger to mediate cellular responses, thus activating immune cells. However, excessive ROS will induce high oxidative stress, promote DNA and protein fatal oxidative damage and membrane lipid peroxidation in AD patients, lead to cell death, and aggravate the disease of AD [15, 16]. In addition, ROS also participates in signal pathways such as NF-κB and p38 MAPK, leading the increase of related proinflammatory cytokines, inducing additional T cell differentiation and macrophage polarization, which is related to the development and deterioration of AD [17, 18]. At the same time, the reactive oxygen species produced by bacterial infection can cause serious damage to blood vessels and endothelial cells, further aggravating AD [19–22]. Therefore, effective microbial elimination and skin surface oxidative stress regulation could be a strategy for the treatment of AD.

Hydrogel with complex network structure is a kind of highly hydrophilic biomaterials, which is widely used as drug carriers for various diseases [23–28]. At the same time, the antibacterial metal organic framework materials (MOFs) are loaded into the hydrogel to regulate the microenvironment of skin damage [29, 30]. MOFs are crystalline porous coordination polymers, which can store and slowly release metal ions, such as zinc, copper and cobalt ions [31, 32]. During the period of antibiotic abuse, ZIF-8 is considered to be one of the most promising types of Zn-MOF, which has been proved to have effective antibacterial activity [33].

**Scheme 1:**
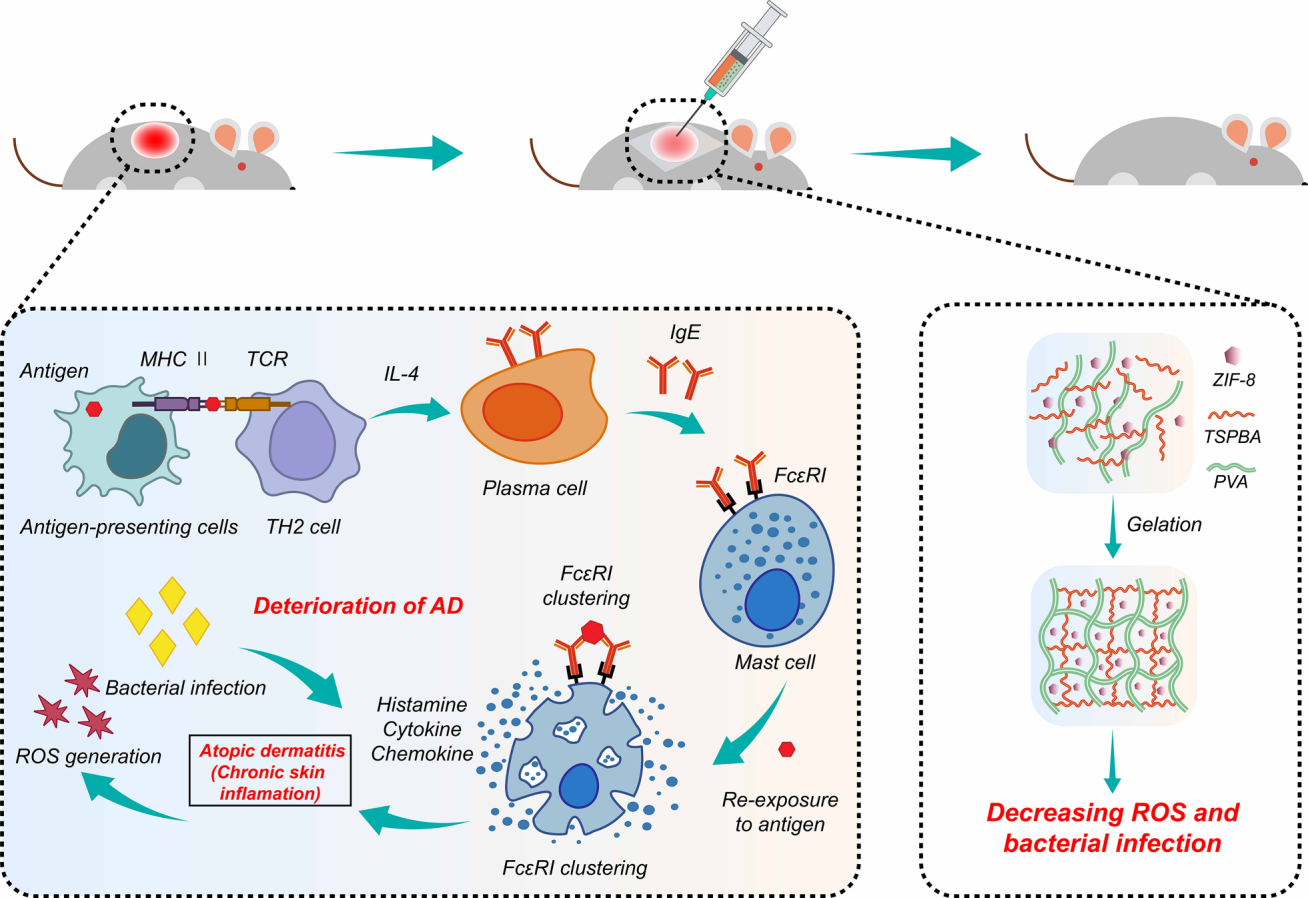
Schematic of the Gel@ZIF-8, which suppresses oxidative stress and decreases inflammatory response

In this study, we encapsulate ZIF-8 in the hydrogel to prepare a PVA based hydrogel (Gel@ZIF-8) with efficient ROS-scavenging ability, good antibacterial effect and high biocompatibility. Here, PVA and ROS response linker (TSPBA) are cross-linked through quaternization reaction to form aryl borate polymer. Aryl borate and its derivatives are common substances for the construction of functional polymers and conjugated molecules. The B-C bond in the structure can be broken to form phenol under ROS response. The hydrogel has good physical properties and biocompatibility, and has an efficient ROS-scavenging ability. At the same time, ZIF-8 is encapsulated in hydrogel, Zn^2+^ released by Zn-MOF can destroy the integrity of bacteria [34]. The rough surface of ZIF-8 nanoparticles can increase the contact area between MOFs and bacteria, which could obtain a better antibacterial effect [35]. In the animal experiment of AD model induced by dinitrochlorobenzene (DNCB), we attach the Gel@ZIF-8 to the diseased skin which shows a good therapeutic effect, such as the reduction of epidermal thickness, the number of IgE and tissue infiltrating mast cells (Scheme 1). Therefore, we believe that the combination of Zn MOF and ROS-scavenging hydrogel provides a promising strategy for AD friendly treatment.

## Methods and materials

### Materials

Polyvinyl alcohol (PVA), N, N, N ', N'–tetramethyl**–**1,3**–**propanediamine and 4–(bromomethyl) phenylboronic acid are purchased from Macklin Company, and other reagents not mentioned are purchased from Aladdin Company. Mouse fibroblasts cells (L929) are cultured in RPMI-1640 culture medium. BALB/c mice are fed according to the scheme approved by the Laboratory Animal Center of Nanjing Tech University, and all animal procedures are in accordance with the regulations of the Animal Protection and Use Committee of Nanjing Tech University.

### Synthesis of ROS-responsive N^1^–(4–boronobenzyl)–N^3^–(4–boronophenyl)–N^1^,N^1^,N^3^,N^3^–tetramethylpropane–1,3–diaminium(TSPBA) linker

TSPBA is synthesized according to previous literature. Briefly, N,N,N′,N′**–**tetramethyl**–**1,3**–**propanediamine and 4**–**(bromomethyl) phenylboronic acid are added into anhydrous N, N**–**dimethylformamide (DMF), and stir them at 60 °C for 16 h to obtain a clear solution. Then, the mixture solution is added into the cold tetrahydrofuran (THF) with a dropper. After the white solid is separated, we centrifugate it for 10 min at the speed of 10000r/min, and wash it three times with THF. After purification, the purified TSPBA is dried and preserved at room temperature for further use. The ^1^H nuclear magnetic resonance (Bruker, AVANCE III, Germany) of TSPBA is obtained by 400 MHz NMR within DMSO.

### Preparation and characterization of ROS-scavenging PVA-TSPBA hydrogel

**Fig. 1:**
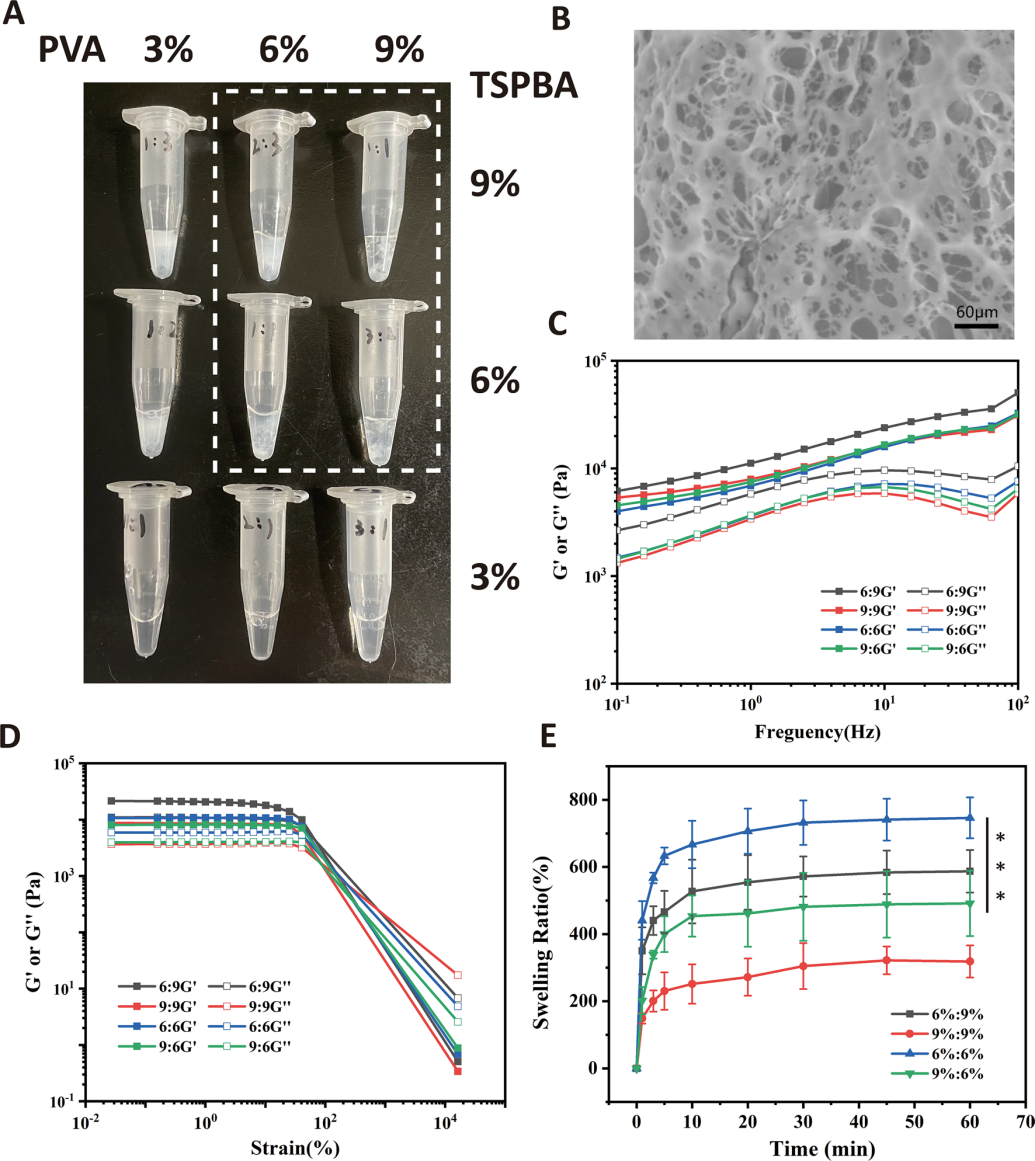
**A** Gel formed with various concentrations of PVA and ROS-sensitive linker (TSPBA). **B** Scanning electron microscopy (SEM) visualization of Gel-3 (6%PVA: 6%TSPBA = 1:1) (scale bar = 60 μm). **C** Frequency spectra and **D** strain spectra of the elastic (G') and viscous (G") moduli of Gel-1 (6%PVA: 9%TSPBA = 1:1), Gel-2 (9%PVA: 9%TSPBA = 1:1), Gel-3 (6%PVA: 6%TSPBA = 1:1) and Gel-4 (9%PVA: 6%TSPBA = 1:1). **E** The swelling ratio of Gel with different concentration ratios (n = 3). *** indicates p < 0.001

Different wt% (3%, 6% and 9%) polyvinyl alcohol (PVA) aqueous solutions and different wt% (3%, 6% and 9%) TSPBA aqueous solutions are mixed by the same volume, and PVA-TSPBA gel(Gel) is prepared (Fig. 1A). After the gel is freeze-dried, Sigma 300 high resolution field emission scanning electron microscope (Hitachi, Regulus 8100, Japan) is used to detect the morphology. We use rheometer (Gooding technology, MCR 301, China) to evaluate the rheological properties of the Gel-3 (6%PVA: 6%TSPBA = 1:1).

### Swelling property test

We cut the Gel-1 (6%PVA: 9%TSPBA = 1:1), Gel-2 (9%PVA: 9%TSPBA = 1:1), Gel-3 (6%PVA: 6%TSPBA = 1:1) and Gel-4 (9%PVA: 6%TSPBA = 1:1) into a 5 mm thick hydrogel with a diameter of 10 mm, freeze dry them, and weigh them (W_0_). Then we put the samples into distilled water, use filter paper to absorb the excess water on the surface of the hydrogel at different times, and record the swelling mass of the hydrogel (W_1_). After recording, we put the hydrogel into distilled water again, and repeat the above operation until the swelling of the hydrogel is balanced. Repeat the same sample for three times, and use the following formula to calculate the swelling ratio (SR) of hydrogels with different mass ratios:

$$SR\%=\frac{\mathrm{W}1-\mathrm{W}0}{\mathrm{W}0}\times 100\%$$

### ROS-scavenging ability evaluation

Gel-3 are immersed in 0 mM, 0.5 mM and 1 mM H_2_O_2_ phosphate buffer saline (PBS) at 37 °C. The morphological changes of hydrogel scaffold are observed at different time periods. Then, the morphology of the hydrogel is observed by SEM at the concentration of 10 mM H_2_O_2_ for 1 h.

Methylene blue (MB) is used as the •OH indicator probe to evaluate the •OH-scavenging ability of the hydrogel. Ferrous ion (Fe^2+^) and H_2_O_2_ solution (1 mM) are used to generate •OH through Fenton reaction. MB (10 μg/mL) is added to the reaction system and incubated with hydrogel. At different times, the absorption of the reaction solution is recorded at 664 nm to calculate the degradation rate of MB.

### Synthesis and characterization of ZIF-8

2-methylimidazole and Zn (NO_3_) _2_ · 6H_2_O are added to methanol, and stirred at room temperature for 15 min. Mix the two solutions, stir them continuously for 1 h to obtain ZIF-8 suspension solution, and wash them with methanol for three times. Remove the supernatant, place the bottom product in a vacuum drying oven, and dry it at 40 °C overnight to obtain white powder ZIF-8 nanoparticles. The size of ZIF-8 is measured by dynamic light scattering with particle size analyzer (Malvern Co. Ltd., Malvern ZEN 3600, UK). The morphology of ZIF-8 is tested by high resolution field emission scanning electron microscope (Zeiss Company, Sigma 300, Germany).

### Preparation of Zn-MOF hydrogel(Gel@ZIF-8)

Firstly, different amounts of ZIF-8 are added to 6 wt% PVA polymer aqueous solution to obtain ZIF-8 suspension, and then mixed with 6 wt% TSPBA aqueous solution to obtain Gel@ZIF-8 (0, 80, 500, and 5000 μ g/mL). Then, SEM is used to observe the morphology of gel.

### Zinc ion release curve of the Gel@ZIF-8

The zinc ion release is measured by spectrophotometry. Firstly, soak the samples with different concentrations in 5 ml of phosphate buffered saline (PBS, pH 7.2–7.4). In this process, the leaching solution is collected and the zinc released from the sample is quantified by zinc spectrophotometry at 620 nm using an enzyme microplate analyzer (Tecan, Infinite 200PRO, Switzerland). After each sampling, add the equal amount of fresh PBS into the container to maintain a constant volume. Then calculate the cumulative release of Zn^2+^ according to the standard curve obtained.

### In vitro cytotoxicity test and cytoprotective test with gel and Gel@ZIF-8 under highly oxidative conditions

Mouse fibroblasts cells (L929) are cultured in RPMI-1640 complete medium containing 10% fetal bovine serum and 1% penicillin/streptomycin, and incubated in a constant temperature incubator at 37 °C and 5% CO_2_. Cells with 5 × 10^3^ density is inoculated on 96 well plate.

For the in vitro cytotoxicity test, after 24 h of cell culture, remove the old culture medium. The Gel@ZIF-8 (the diameter is 5 mm, the thickness is 1 mm) are added to the surface, and the culture is still under the condition of 37 °C, 5% CO2. Add the same amount of RPMI 1640 complete medium as the blank control. After 24 h, remove the Gel@ZIF-8, and stain the cells with calciflavone and propidium iodide (Yesen Biotechnology Co., Ltd.), and observe the cell viability with fluorescence microscope (Nikon, TS100-F, Japan). After 24 h and 48 h, the cell viability is measured at 490 nm OD by MTT method.

For cytoprotective tests with hydrogen peroxide, cells with 5 × 10^3^ density is inoculated on 96 well plate. After 24 h, when Gel and Gel@ZIF-8 exist, add 200 μL RPMI-1640 medium containing 1000 μM H_2_O_2_. After 24 h, and the cell viability is determined by MTT method.

### Antibacterial ability test

The antibacterial activity of Gel@ZIF-8 is determined by plate colony count method. Take the bacterial solutions of S.aureus, MRSA and E.coli respectively, and add different concentrations of Gel@ZIF-8. After incubation in a 220 rpm shaking table at 37° C for 2 h, the cocultured bacterial solution was diluted with PBS in a series of gradients, incubating on a shaker for 24 h (37 °C, 50 rpm). 100μL diluted bacterial suspension is dropped on the agar plate, inverted in the mold incubator at 37 °C for 24 h. Count the number of bacterial colonies on the agar plate, and calculate the antibacterial rate. Each experiment is conducted three times and the average value is calculated.

### In vivo therapeutic effect of CeNP hydrogel DNCB-induced AD mouse model

Six-week-old BALB/c mice are used for in vivo experiments. The mice are randomly divided into 5 groups (n = 6). All animal experiment procedures are approved by the Experimental Animal Center of Nanjing Tech University. One day before the experiment, the dorsal hair of all mice are shaved to approximately 4cm^2^. DNCB is used to induce back atopic dermatitis. A certain amount of DNCB is dissolved in a 3:1 mixture of acetone and olive oil. In the previous week, 150μL 2% (v/v) DNCB is applied to the dorsal segment of each mouse. Then, 150μL 0.5% (v/v) DNCB is used to sensitize the back skin of mice twice every two days until the 14th day. During the experiment, apply the dexamethasone cream (DXMS), Gel and Gel@ZIF-8 once every two days.

During the entire study, the dermatitis score was measured as the sum of scores graded as 0 (none), 1 (mild), 2 (moderate), or 3 (severe) for each of the four representative symptoms of atopic dermatitis (erythema/hemorrhage, scarring/dryness, edema, and excoriation/erosion). After finishing the study, all mice were sacrificed. The middle site of the dorsal segment is collected and fixed in formalin solution for histological analysis. Blood is collected from hearts during sacrifice. The samples are stained with H&E and toluidine blue, and the images are analyzed using an optical microscope (Ti-U, Nikon, Japan). The epidermal thickness is measured from each image of H&E staining by Image J. The number of mast cells is counted in each image using toluidine blue staining. The serum is separated and stored at −80 °C before use. The levels of IgE in serum are measured using enzyme-linked immunosorbent assay. All experiments are performed according to the manufacturer’s instructions.

### Statistical analysist

All values are presented as the mean ± SD as the error bar. Statistical analysis is performed by one-way ANOVA for multiple comparisons followed by tukey posthoc test or two-tailed Student’s t-test using Origin software. In all cases, statistical significance is set at *p < 0.05, **p < 0.01, ***p < 0.001.

## Results and discussion

### Preparation and characterization of ROS-scavenging PVA-TSPBA hydrogel

Firstly, ROS responsive linkers N^1^–(4–boronobenzyl)–N^3^–(4-boronophenyl)–N^1^, N^1^, N^3^, N^3^–tetramethylpropane–1,3–diaminium (TSPBA) are synthesized by quaternization reaction from N, N, N', N'-Tetramethyl-1,3-propanediamine and 4–(bromomethyl) phenylboronic acid. The structure of TSPBA is confirmed with ^1^H NMR (400 MHz, DMSO) (Additional file 1: Fig. S1).

Then, we study the effect of the concentration of polyvinyl alcohol (PVA) and TSPBA linker on the gel formation (Fig. 1A). Through simple mixing of wt% (3%, 6% and 9%) PVA aqueous solution and wt% (3%, 6% and 9%) TSPBA aqueous solution, ROS responsive hydrogel (Gel) can be rapidly formed by phenylboric acid and alcohol hydroxyl. When TSPBA concentration is high (more than 6%), solid gel can be formed regardless of PVA concentration. The PVA concentration may be relatively low (3%), and the local concentration of TSPBA is high during the reaction, the colloid formed is easy to disperse into blocks, and there is no good integrity after freeze-drying. For this reason, we chose four groups of hydrogels with PVA and TSPBA ratios of Gel-1 (PVA: TSPBA = 6%:9%), Gel-2 (PVA: TSPBA = 9%:9%), Gel-3 (PVA: TSPBA = 6%:6%) and Gel-4 (PVA: TSPBA = 9%:6%) for subsequent experiments.

After the prepared gel is freeze-dried, through the scanning electron microscope (SEM), we can clearly observe that the surface of polyvinyl alcohol-based antioxidant hydrogel (Gel-3) is rough, and the hydrogel presents a porous microstructure with interpenetrating networks, and the pore distribution is dense, with the pore size ranging from 20 μm to 60 μm (Fig. 1B).

Rheological tests further confirm the formation of hydrogel. The rheological properties of PVA based hydrogels are characterized by monitoring their storage modulus (G ') and loss modulus (G ") as a function of frequency and stress. For frequency scanning (Fig. 1C), all tested gel show similar nonlinear rheological behavior, and their values increase with the increase of frequency, which means that there is similar microstructure. In addition, the tangent value of the loss angle represents the ratio between the viscous and elastic properties, and is a sensitive indicator of the motion of various molecules within the material, with tan δ = G "/G'. The lower the value of tanδ, the more elastic the material, where a value of tanδ < 1 usually indicates that the sample is elastic, while a value of tanδ > 1 corresponds to a viscous sample. The loss angle tangent values of Gel-1 (6–9%), Gel-2 (9–9%), Gel-3 (6–6%) and Gel-4 (9–6%) were 0.409, 0.301, 0.417 and 0.373, respectively. All of these values are less than 1, indicating that all samples are elastic. For strain scanning (Fig. 1D), the G' and G" values of the four groups of hydrogels decrease with the increase of strain, indicating the dissociation of chemical bond crosslinking and the collapse of network structure. In the linear viscoelastic region, the value of G' is higher than G", indicating that the hydrogel is a viscoelastic solid.

### Swelling property

Swelling rate (SR) is an important parameter of hydrogel. High SR is conducive to maintaining a moist wound environment and improving inflammation, so hydrogel is required to have good swelling performance. As shown in the Fig. 1E, in the first 10 min of the swelling test, the swelling ratio of all hydrogels with different concentration ratios has increased. Among them, the maximum swelling ratio of Gel-3 is close to 750%, and with the increase of the concentration of PVA or TSPBA, the swelling ratio of the other three groups of hydrogel gel decreases, of which Gel-2 hydrogel has the lowest swelling ratio. According to the above experimental results, Gel-3 (6%PVA: 6%TSPBA = 1:1) is selected for subsequent experiments.

The prepared hydrogel can quickly reach the expansion equilibrium, which may be caused by the wicking effect of PVA-TSPBA hydrogel pores, and the existence of a large number of hydroxyl groups (–OH) in the molecular chain of PVA, which can form hydrogen bonds with water molecules, locking a large number of water molecules in three-dimensional porous structure. As the concentration ratio increases, the hydrogel crosslinks become a more compact structure, the pore size of the three-dimensional porous structure decreases, and water molecules are not easy to diffuse in the narrow pores, resulting in a lower swelling behavior, which further limits the network to absorb more water. Therefore, the prepared hydrogel can maintain the moist microenvironment of the injured part, and can help reduce the dryness of atopic dermatitis.

### ROS-scavenging ability of gel

**Fig. 2:**
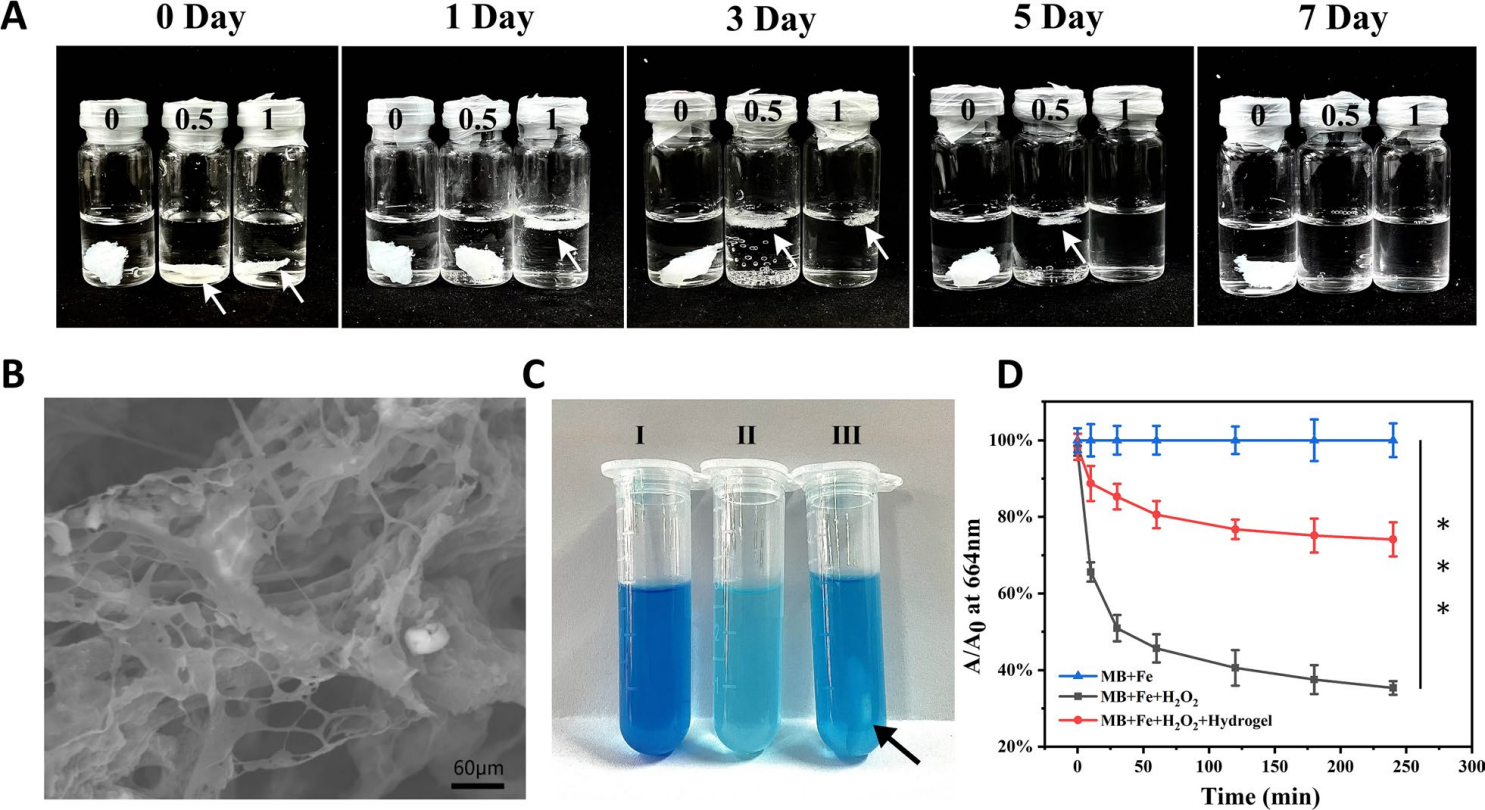
**A** The changes of Gel in H_2_O_2_ (0 mM, 0.5 mM and 1 mM) at different times. **B** Scanning electron microscopy (SEM) visualization of Gel in 10 mM after 1 h (scale bar = 60 μm). **C** The photo of MB in Fenton reaction solution incubates with or without the hydrogel (I: MB + Fe, II: MB + Fe + H_2_O_2_, III: MB + Fe + H_2_O_2_ + Hydrogel). The place pointed by the black arrow is the hydrogel. **D** The relative absorbance value of MB triggered by Fenton reaction with or without the hydrogel. (n = 3). *** indicates p < 0.001

Because aryl borate esters and their derivatives are common materials for building functional polymers and conjugated molecules, the B-C bond in their structures can be broken to generate phenol under the action of H_2_O_2_, and can become one of the biodegradable materials. In order to explore the response degradation ability of the hydrogel prepared under H_2_O_2_ environment, the hydrogel is incubated with H_2_O_2_ to observe the changes at different times. As shown in the Fig. 2A, with the increase of H_2_O_2_ concentration and reaction time, the hydrogel response speed increases and the time required for degradation decreases. The morphology of the hydrogel incubated with H_2_O_2_ is further observed by SEM (Fig. 2B). Under the action of 10 mM H_2_O_2_, the porous structure of the hydrogel is destroyed after 1 h. This shows that the hydrogel prepared has good response to ROS.

The scavenging activity of hydrogel against hydroxyl radical (•OH) is studied through using methylene blue (MB) as the •OH indicator probe. As shown in the Fig. 2C, ferrous ion (Fe^2+^) and 1 mM H_2_O_2_ solution are used to generate •OH through Fenton reaction. The color of MB solution quickly changes from dark blue to light blue, indicating the generation of •OH. However, after adding hydrogel, the color change of MB in •OH solution is small. As shown in the Fig. 2D, with the increase of time, the relative absorbance gradually decreases, showing a dependence on time. After 240 min, the relative absorbance value of the hydrogel group decreases by about 25%, and that of the anhydrous gel group decreases by about 65%. It can be seen that the hydrogel prepared has a good response to • OH, and it also shows that our hydrogel has a strong ability to scavenge • OH.

### Synthesis and characterization of ZIF-8 nanoparticles

**Fig. 3:**
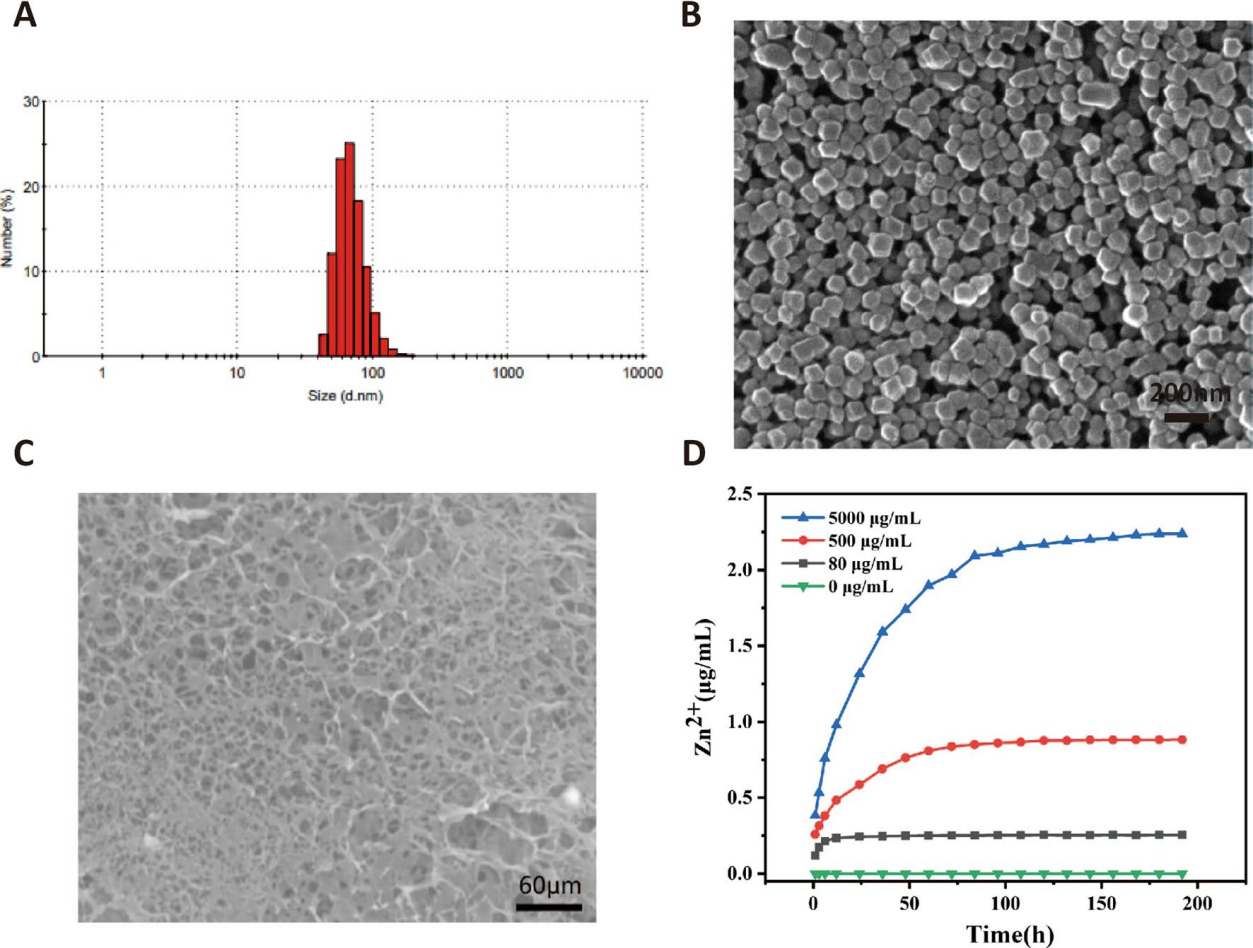
**A** Particle size distribution of ZIF-8. **B** The morphology and size of ZIF-8 nanoparticles verified by SEM. **C** Scanning electron microscopy (SEM) visualization of Gel@ZIF-8 (scale bar = 60 μm). **D** Cumulative release profile of Zn^2+^ from Gel@ZIF-8 (n = 3). ** and *** indicate p < 0.01 and p < 0.001, respectively

The ZIF-8 nanoparticles consist of 2-methylimidazole (2-melm) and zinc nitrate hexahydrate (Zn (NO_3_) _2_) via covalent bonds. The average particle size and dispersion index PDI of ZIF-8 are detected by the particle size analyzer. The particle size of ZIF-8 is normally distributed, with an average particle size of 98.72 nm and a PDI of 0.096, showing a good control over the size of nanoparticles (Fig. 3A). The morphology and size of ZIF-8 nanoparticles verified by SEM show that they have a landmark hexagonal structure (Fig. 3B). The X-ray diffraction test results are shown in Additional file 1: Fig. S2. There are obvious strong peaks at 2θ = 7.26°, 10.33°, 12.68°, 14.63°, 16.88° and 18.11°, corresponding to crystal plane (011), (022), (112), (022), (013) and (222) respectively. The characteristic peaks of the prepared ZIF-8 are consistent with the simulated ZIF-8XRD pattern (JCPDS No: 10–0454), indicating that ZIF-8 has been successfully synthesized and has high crystallinity. When ZIF-8 is decomposed, the released Zn^2+^ coordinates with the hydroxyl group on the hydrogel molecule, which reduces the voids in the hydrogel network and makes the pore distribution and shape more compact and regular (Fig. 3C).

### Zinc ion release curve of the Gel@ZIF-8

Under physiological conditions, Zn^2+^ is usually used as a booster for human growth and immunity. However, high concentration of Zn^2+^ has potential toxicity to cell growth. In order to evaluate the release kinetics of Zn^2+^ after decomposition of Zn MOF, a series of standard curves (Additional file 1: Fig. S3) of Zn^2+^ concentration gradient are established by zinc spectrophotometry method, color blocks represent the colors of solutions with different concentrations. The release amount of Zn^2+^ is positively correlated with the concentration of ZIF-8 nanoparticles, showing a steady upward trend within a week (Fig. 3D).

### In vitro cytotoxicity test and cytoprotective test with Gel and Gel@ZIF-8 under highly oxidative conditions

**Fig. 4:**
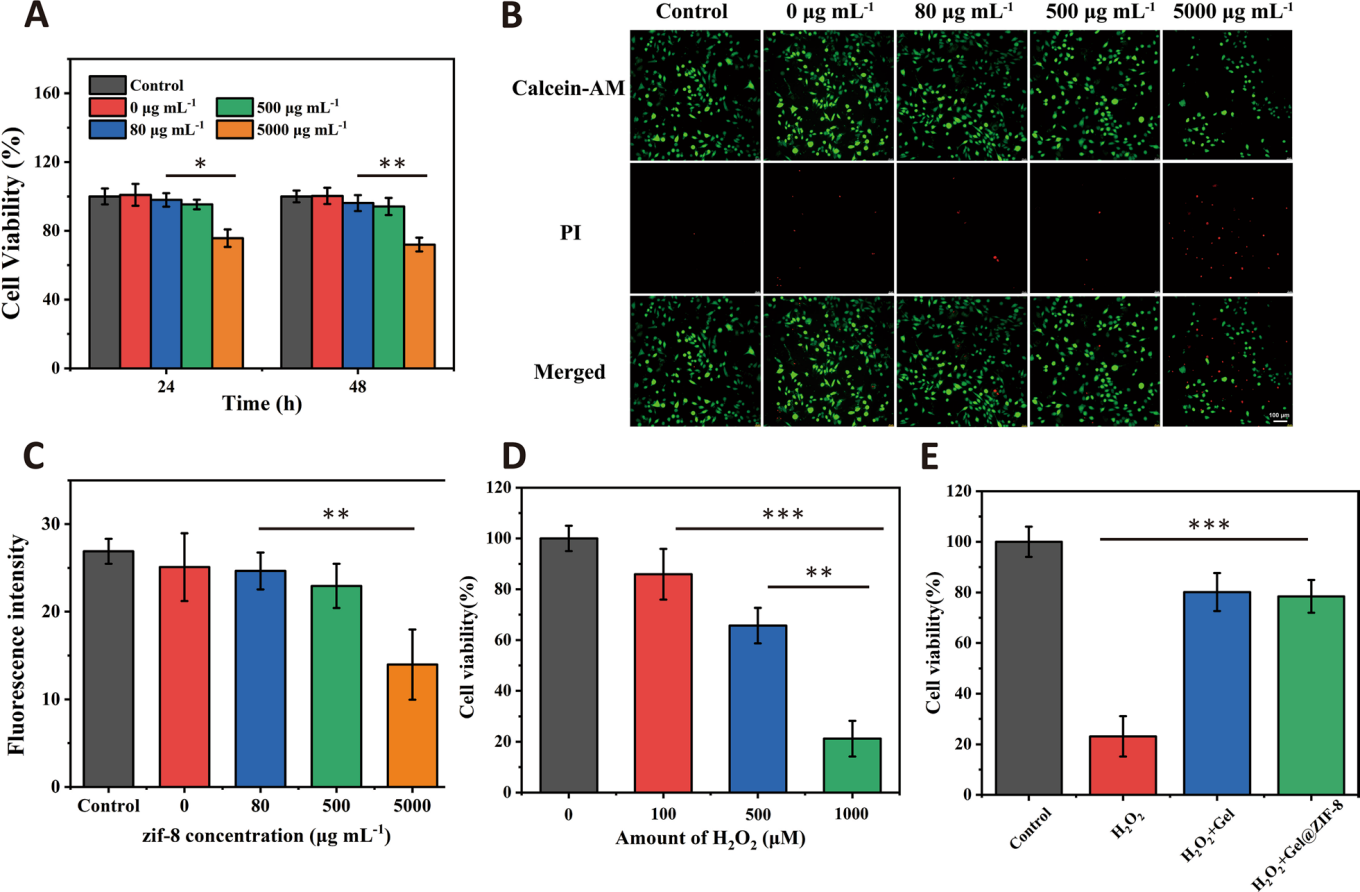
**A** Cell viability of different Gel@ZIF-8 on L929 cells at 24 h and 48 h. **B** The photo of live/dead cell staining at 48 h. (scale bar = 100 μm). **C** Fluorescence intensity of the live/dead cell staining at 48 h. **D** Cell viability of L929 cells with various H_2_O_2_ concentrations. **E** Cytoprotective effect of Gel@ZIF-8 under a highly oxidative medium (H_2_O_2_) (n = 3). *, ** and *** indicate p < 0.05, p < 0.01 and p < 0.001, respectively

The presence of ZIF-8 and the release of zinc ions may cause toxicity to cells. Therefore, we use different concentrations of Gel@ZIF-8 to coculture with L929 cells to evaluate the cytotoxicity of the Gel@ZIF-8. MTT assay and live/dead cell staining are used to detect cell viability. As shown in the Fig. 4A, the OD value is measured by MTT method at 490 nm. When the concentration of ZIF-8 nanoparticles exceeds 500 μg/mL, the proportion of living cells decreases, which inhibit the cell growth. In addition, at concentrations of 0, 80, 500 and 5000 μg/mL, the number of living cells assessed by live/dead cell staining is basically the same (Fig. 4B and C).

In order to test the protective ability of Gel@ZIF-8 on cells, L929 cells are placed in H_2_O_2_ (Fig. 4D). Gel or Gel@ZIF-8(the diameter is 5 mm, the thickness is 1 mm) are added in 1000 μM H_2_O_2_ at the same time (Fig. 4E). Compared with the control group, the addition of Gel and Gel@ZIF-8 significantly improve the cell viability, indicating that Gel@ZIF-8 can prevent cell damage by scavenging excessive H_2_O_2_ from the culture medium.

### Antibacterial ability

**Fig. 5:**
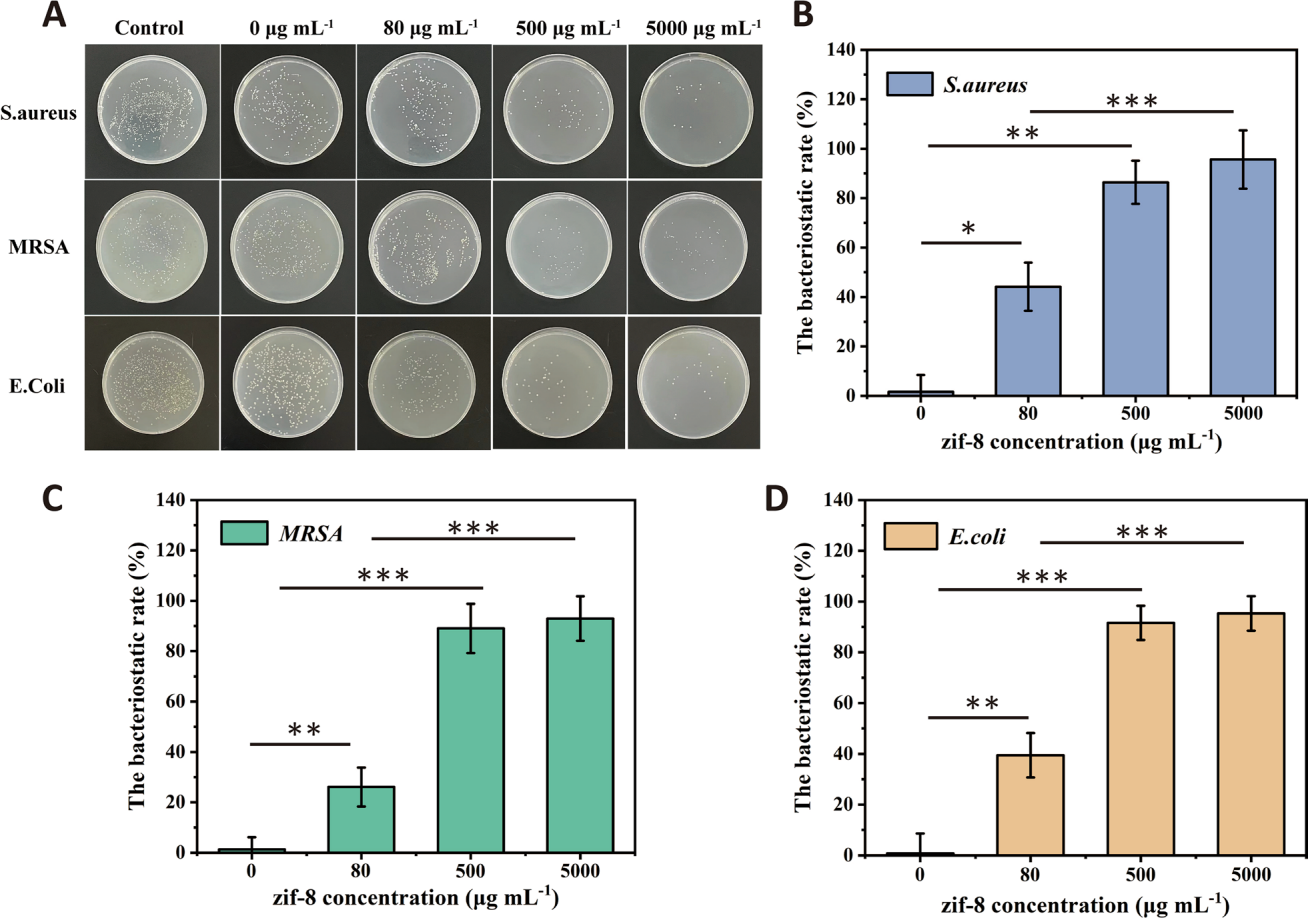
**A** The statistical graph of the bacteriostatic rate of the Gel at different ZIF-8-laden concentrations. The bacteriostatic rate of **B** S. aureus **C** MRSA **D** *E. coli* (scale bar = 100 μm, n = 3). *, ** and *** indicate p < 0.05, p < 0.01 and p < 0.001, respectively

Atopic dermatitis is easy to be infected by malignant bacteria such as Staphylococcus aureus, which aggravates inflammation and even presents life-threatening complications. Therefore, preventing bacterial infection is the key to the treatment of dermatitis. We evaluate the bactericidal activity of Gel@ZIF-8. ZIF-8 can remove microorganisms and pathogens harmful to inflammatory skin lesions. ZIF-8 can inhibit microorganisms and pathogens harmful to inflammatory skin lesions. We study the bactericidal properties of 0、80、500 and 5000 μg/mL Gel@ZIF-8. As shown in the Fig. 5A, after 24 h of incubation, the higher the concentration of ZIF-8 nanoparticles, the fewer colonies of S. aureus, MRSA and E. coli. The bacteriostatic rate increases significantly (Fig. 5B–D), According to the cell experiment results, Gel has no direct effect on microorganisms and pathogens, and the appropriate concentration of ZIF-8 nanoparticles is 500 μg/mL, which has good cell biocompatibility and antibacterial effect.

### In vivo therapeutic effect of Gel@ZIF-8 DNCB-induced AD mouse model

**Fig. 6:**
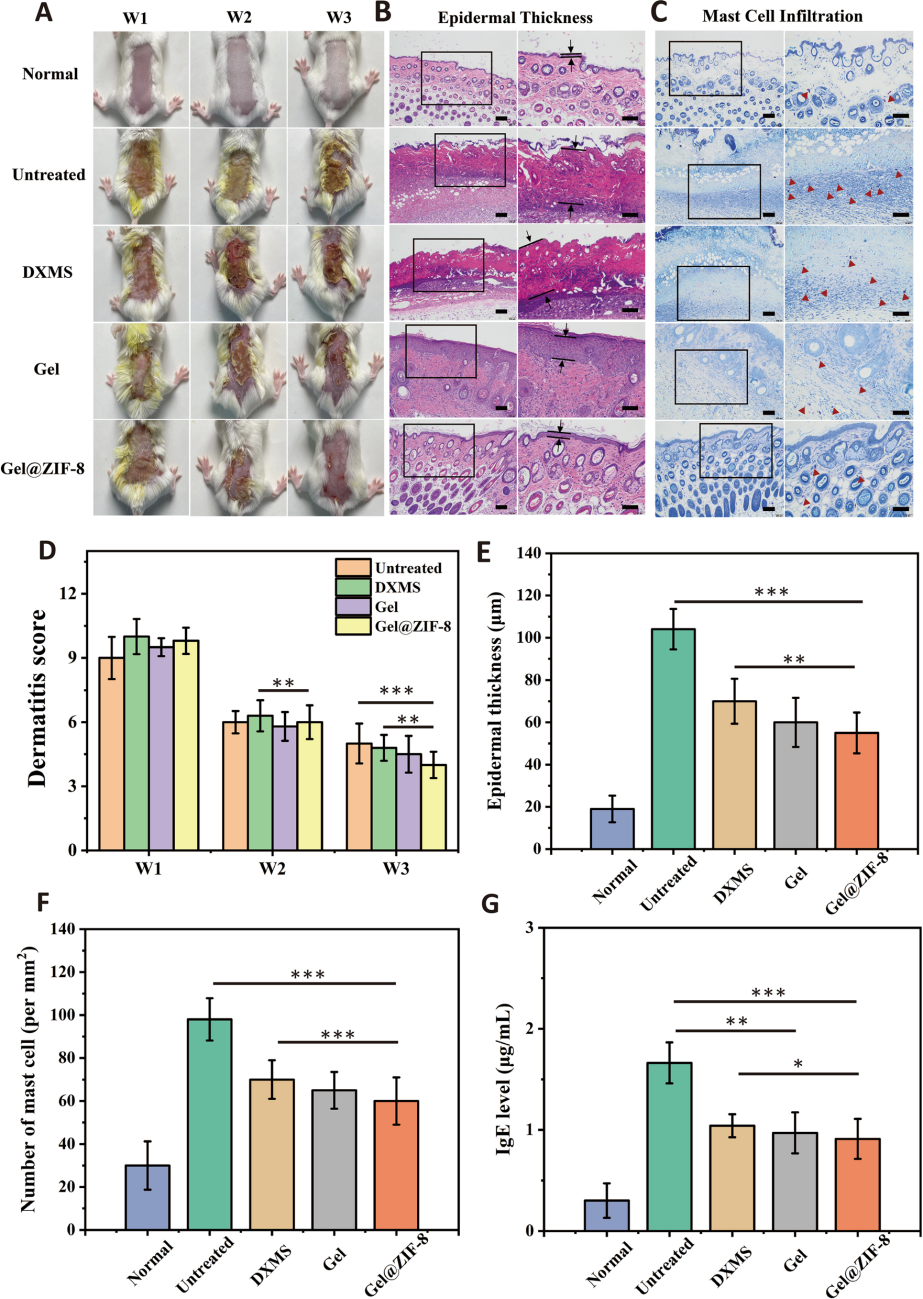
**A** Representative photographs of dorsal skin of each group for monitoring the change in the lesion. **B** Histology of mouse skin sections stained with H&E. The space between red lines denotes the epidermal thickness. **C** Histology of mouse skin sections stained with toluidine blue for dermal mast cells. The red arrow heads indicate the mast cells (scale bar = 100 μm). **D** Dermatitis score measurements conducted over three weeks. **E** Comparison of epidermal thickness. **F** Measurement of the number of mast cells for each group. **G** The concentrations of IgE in blood serum retrieved from each group at W3. (n = 6). *, ** and *** indicate p < 0.05, p < 0.01 and p < 0.001, respectively

To investigate the therapeutic potential of the Gel@ZIF-8 (500 μg/mL) for the treatment of AD, a mouse AD model is induced with DNCB. DNCB is one of the chemicals used to prepare AD animal models. When applied to skin, DNCB interacts with skin protein to form a complex, which is absorbed by antigen presenting cells, and then activated Th2 cells and mast cells. Figure 6A shows that the skin of mice treated with DNCB contains a compound of blood and pus, indicating that AD is well induced in the skin in the first week.

After 14 days of treatment, the skin of untreated group, dexamethasone group and Gel group still have wounds, while the skin of ZIF-8 hydrogel group has smaller wounds. The thickness of epidermal layers is a representative indicator of AD. The untreated groups, DXMS and Gel groups show epidermal layers 5.3, 3.5 and 3.0-fold thicker than the healthy group, respectively. The Gel@ZIF-8 groups recover a thinner epidermal thickness, which is twofold smaller than that in the untreated group (Fig. 6B, E). A large number of mast cells is a characteristic feature of AD, so mast cells are visualized by toluidine blue staining. The results reveal that the lowest infiltration of mast cells in the dermis is in the Gel@ZIF-8 group (Fig. 6C, F). The dermatitis scores show that the diseases are provoked with similar severity in all mice at week 1, and the score decreases to different degrees depending on the treatment (Fig. 6D). The score changes of untreated group, dexamethasone group and blank hydrogel group are similar, while the dermatitis score of ZIF-8 hydrogel group is the lowest, with a statistically significant difference.

We further evaluate the changes of AD related immune protein levels after hydrogel treatment. IgE is a representative biomarker of AD, which can enhance mast cell activation, allergen internalization and other immune responses. It can be seen from the Fig. 6G that the IgE level in the blood of AD mice in Gel group decreases, and the Gel@ZIF-8 continues to produce therapeutic effect.

Thus, after treating the skin of AD mice, Gel@ZIF-8 group can reduce the size of AD skin wound, restore the thickness of epidermis, and inhibit AD related immune factors, including mast cell infiltration and IgE.

## Conclusion

Atopic dermatitis (AD) is a kind of chronic recurrent skin inflammation. High levels of ROS and bacterial infection generated continuously during the pathological process will destroy the homeostasis of immune response, thus worsening the AD state. In this study, a Zn-MOF oxidative stress hydrogel is proposed to alleviate the imbalance of immune response and regulate the damage microenvironment by eliminating ROS and inhibiting bacterial infection. The hydrogel shows high ROS removal efficiency and has a good protective effect on cells. The antibacterial agent ZIF-8 loaded into the hydrogel can reduce its toxic and side effects and show a lasting and effective antibacterial activity. In animal experiments, Gel@ZIF-8 reduces the thickness of mouse epidermis and the number of mast cells and IgE antibodies, promotes the regeneration of inflammatory tissues, and enhances the recovery of mouse skin induced by AD. The results show that the hydrogel can improve AD by locally regulating the inflammatory skin damage microenvironment, which provides a new strategy for the treatment and management of AD.

## Supplementary Information

**Additional file 1: Fig. S1.** ^1^H-NMR (400 MHz, in DMSO) spectrum of TSPBA. **Fig. S2.** XRD patterns of the ZIF-8 collected after reaction. **Fig. S3.** A Zn^2+^ standard curve measured by a spectrophotometric method.

## Data Availability

This published article includes all data generated and analyzed during this research.
