# Supplementary material for: Zn-MOF hydrogel: regulation of ROS-mediated inflammatory microenvironment for treatment of atopic dermatitis
Source: J Nanobiotechnology. 2023 May 22;21:163. doi: 10.1186/s12951-023-01924-0 (PMC10204188; doi:10.1186/s12951-023-01924-0)
Supplement: Supplementary file 1 — Additional file 1: Fig. S1. 1H-NMR (400 MHz, in DMSO) spectrum of TSPBA. Fig. S2. XRD patterns of the ZIF-8 collected after reaction. Fig. S3. A Zn2+ standard curve measured by a spectrophotometric method. [file 12951_2023_1924_MOESM1_ESM.docx]

**Additional Information**

Zn-MOF hydrogel: Regulation of ROS-mediated inflammatory microenvironment for treatment of atopic dermatitis


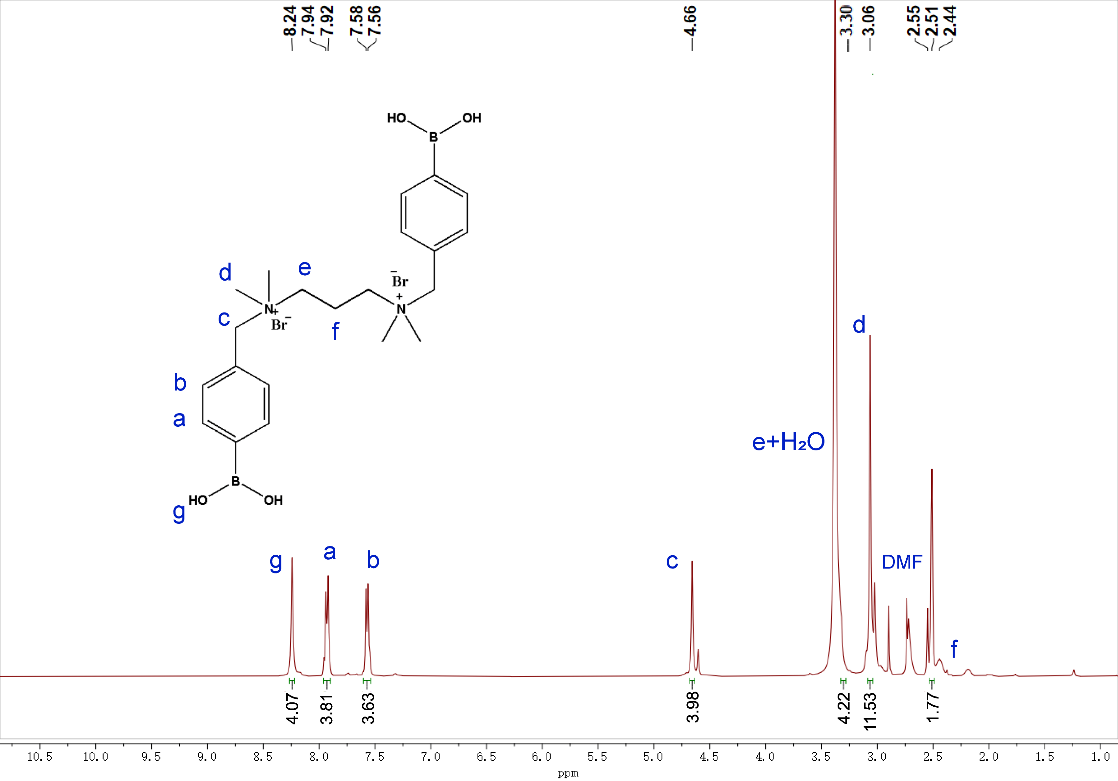


**Fig. S1.** ^1^H-NMR (400 MHz, in DMSO) spectrum of TSPBA.


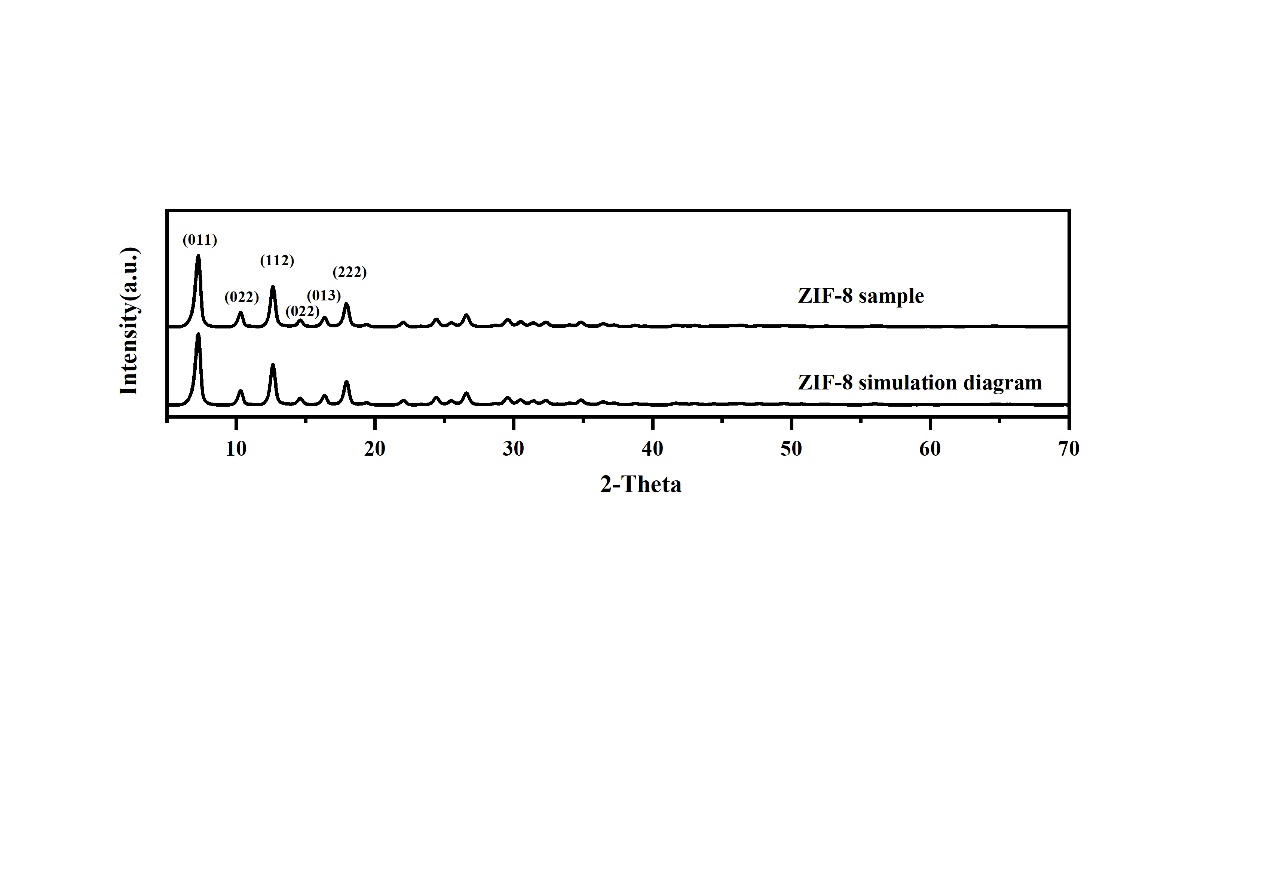


**Fig. S2.** XRD patterns of the ZIF-8 collected after reaction.


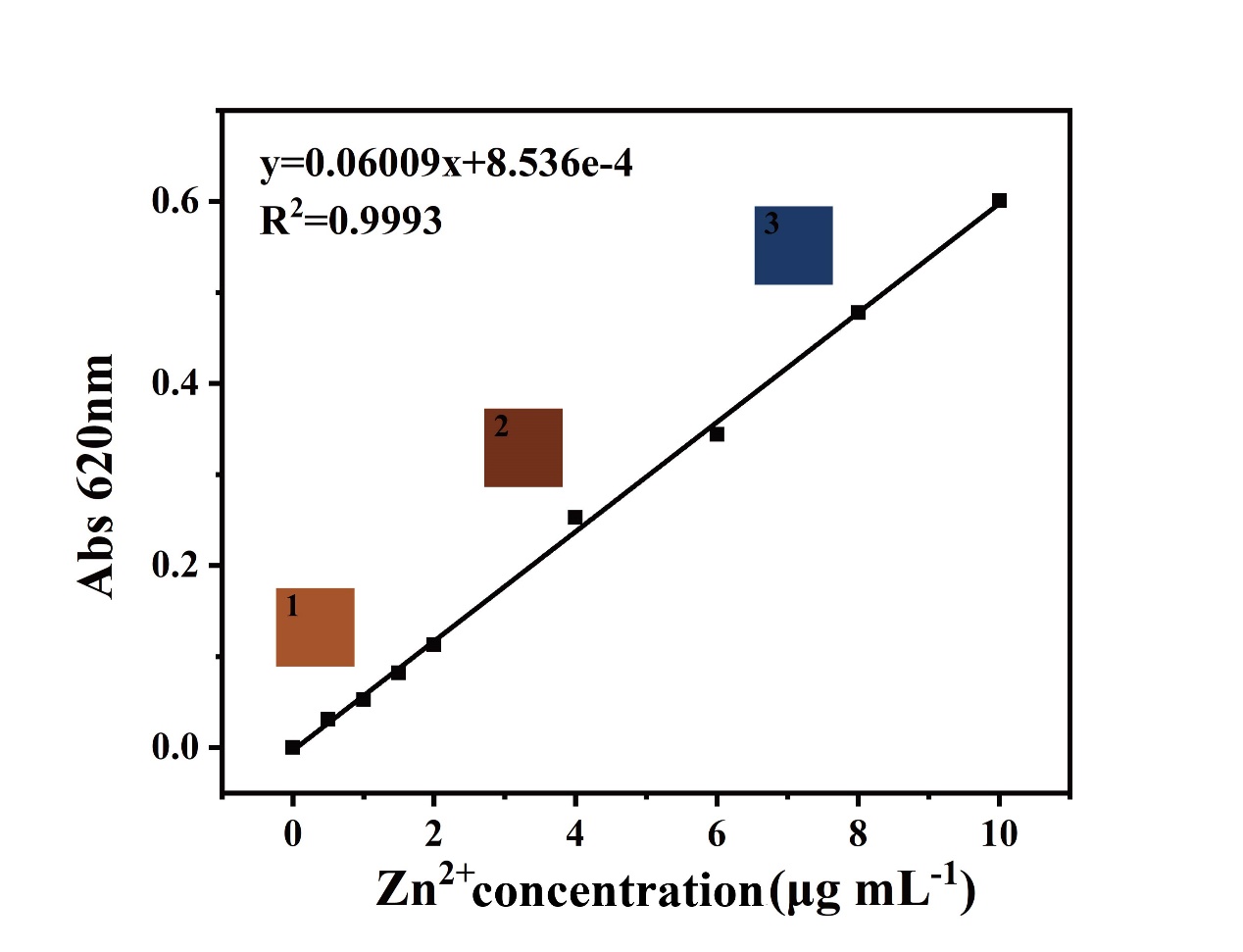


**Fig. S3.** A Zn^2+^ standard curve measured by a spectrophotometric method.
